# *Schistosoma mansoni* egg-derived extracellular vesicles: A promising vaccine candidate against murine schistosomiasis

**DOI:** 10.1371/journal.pntd.0009866

**Published:** 2021-10-13

**Authors:** Shereen F. Mossallam, Iman F. Abou-El-Naga, Amany Abdel Bary, Eman A. Elmorsy, Radwa G. Diab

**Affiliations:** 1 Department of Medical Parasitology, Faculty of Medicine, Alexandria University, Alexandria, Egypt; 2 Department of Pathology, Faculty of Medicine, Alexandria University, Alexandria, Egypt; James Cook University Division of Tropical Health and Medicine, AUSTRALIA

## Abstract

Extracellular vesicles (EVs) are protein-loaded nano-scaled particles that are extracellularly released by eukaryotes and prokaryotes. Parasite’s EVs manipulate the immune system, making them probable next-generation vaccines. Schistosomal EVs carry different proteins of promising immunizing potentials. For evaluating the immune-protective role of *Schistosoma mansoni* (*S*. *mansoni*) egg-derived EVs against murine schistosomiasis, EVs were isolated from cultured *S*. *mansoni* eggs by progressive sequential cooling ultra-centrifugation technique. Isolated EVs were structurally identified using transmission electron microscope and their protein was quantified by Lowry’s technique. Experimental mice were subcutaneously immunized with three doses of 20 μg EVs (with or without alum adjuvant); every two weeks, then were challenged with *S*. *mansoni* cercariae two weeks after the last immunizing dose. Six weeks post infection, mice were sacrificed for vaccine candidate assessment. EVs protective efficacy was evaluated through parasitological, histopathological, and immunological parameters. Results showed significant reduction of tegumentally deranged adult worms, hepatic and intestinal egg counts reduction by 46.58%, 93.14% and 93.17% respectively, accompanied by remarkable amelioration of sizes, numbers and histopathology of hepatic granulomata mediated by high interferon gamma (IFN γ) and antibody level. Using sera from vaccinated mice, the molecular weight of EVs’ protein components targeted by the antibody produced was recognized by western immunoblot. Results revealed two bands of ~ 14 KDa and ~ 21 KDa, proving that EVs are able to stimulate specific antibodies response. In conclusion, the present study highlighted the role of *S*. *mansoni*-egg derived EVs as a potential vaccine candidate against murine schistosomiasis mansoni.

## Author summary

All types of cells were proved to secrete nano-spheres called extracellular vesicles (EVs). Parasites derived-EVs have the ability to manipulate the immune system, and carry many proteins that have been used as a vaccine candidate. In this study, the author used EVs derived from cultured *Schistosome mansoni* eggs as an immunizing agent against experimental schistosomiasis. Mice were immunized with three subcutaneous doses of EVs. Two weeks after the last vaccination dose mice were exposed to cercarial challenge. Six weeks later mice were sacrificed and vaccine efficacy was evaluated using different parasitological, histopathological and immunological parameters. Results showed significant reduction in the parasite burden especially the hepatic and intestinal egg count. Adults obtained from the vaccinated mice showed sever derangement in their protective tegumental layer with loss of muscle tone when examined by electron microscope. EVs induced high level of protective IFN γ and antibody. This was accompanied by amelioration of the hepatic histopathology which is the main cause of the clinical manifestation.

## 1. Introduction

Genus *Schistosoma*, the causative agent of schistosomiasis, is endemic in many tropical and subtropical countries, mainly in poor communities. It is estimated that schistosomiasis infects 230–250 million people annually leading to approximately 280,000 deaths per year. Nearly 258 million people are infected worldwide, with up to 779 million at risk of being infected [1].

During acute illness, the predominant immune response to schistosomula antigens is skewed towards T helper 1(Th1) cytokines, and as the disease progresses, this is shifted to a Th2 response caused by egg-antigens [2]. Chronic morbidity in *Schistosoma mansoni* (*S*. *mansoni*) infection is mainly caused by *Schistosoma* eggs that lodge in the liver and gut causing extensive tissue damage. Host mounts a strong immunological response to parasite eggs forming a collagen-rich granulomatous reaction around the eggs. Although this reaction sequesters antigenic egg products, it leads to severe hepatic fibrosis and portal hypertension [3].

Treatment of schistosomiasis depends solely on praziquantel, which started to encounter the emergence of parasite with reduced sensitivity both in the field and laboratory [4,5]. In spite of mass drug administration programs, re-infections go far beyond the capacity of these programs due to the unchanged people’s activities and continuous cercarial exposure. Also, cessation of drug treatment led to reappearance of the disease in areas that were secure [5]. Thus, vaccines, and/or chemotherapy combined with vaccination, would probably present the best strategies for long-term sustained control of schistosomiasis [6].

Different anti-schistosomal vaccine strategies have been studied. The highest levels of protection have been afforded by attenuated cercarial vaccine [7]. Second generation vaccine candidates depend on using molecules that play a functional role in parasite homeostasis. Multivalent vaccine formulation containing promising antigens have shown promising results as they might enhance subunit vaccine efficacy [8]. Out of many studied vaccine candidates, only three *Schistosoma* antigens have reached clinical trials; fatty acid-binding protein of 14 kDa from *S*. *mansoni* [9], glutathione-S-transferase of 28 kDa from *S*. *haematobium* [10] and *S*. *mansoni* tetraspanin-2 (TSP-2) of 23 KDa [11]. Studies on anti-schistosome vaccine are still on going.

Extracellular vesicles (EVs) are membrane-bound vesicles, released by both eukaryotic and prokaryotic cells, have promising therapeutic, diagnostic and immunizing applications in various medical fields [12]. EVs transmit information between cells, organs and even between organisms; through a range of bioactive macromolecules (protein, mRNA, miRNA) and immunogenic moieties that can effectively change the biological properties of target cells [12,13]. They have important roles in intercellular signalling and waste management. They contribute to important biological functions such as tissue repair, neural communication, transfer of pathogen proteins, and contribute to diseases such as cancer and neurodegeneration. Circulating tumour-derived EVs have emerged as promising cancer progression bio-markers and as novel targets for future anticancer therapies [12].

EVs’ protective roles against different helminthic infections such as, *Opisthorchis viverrini*, *Trichuris*, *Echinostoma*, and *Heligmosomoides* have been proven [14–17]. Parasites transfer their biological cargo to host cells by secreting EVs that can modulate the immune response, hence parasitic EVs are implicated as possible vaccine candidates [18]. Some proteins that have been proven as vaccine candidates against different helminthic infections have recently been found in the EVs of different helminthic parasites [13,19,20]. Parasite-derived EVs contain proteins homologous to the parasitic stage from which they have been derived [13]. Also, EVs carry some protein homology to other developmental stages of the parasite [20].

In schistosomiasis, EVs vaccine studies have been conducted against different species and stages, yet focusing on EVs derived from the adult stage of the parasite [13,21,22]. About one third of proteins found in *S*. *mansoni* adult worm EVs are homologues to previously described vaccine candidates [22]. Some of these proteins are shared between different developmental stages of the parasite [22]. Studies on the egg stage are limited to that performed on *S*. *japonicum* egg-derived EVs, proving their in vitro ability to transfer their cargo to recipient cells, hence demonstrating the regulatory role of schistosomal egg EVs in host parasite interaction [23]. Internalization of schistosomal EVs cargo proved to modulate the immune response. This suggests the possibility of using schistosomal eggs-derived EVs as possible vaccine candidate [24]. Accordingly, the present work has been conducted to evaluate, for the first time EVs derived from *S*. *mansoni* eggs as a potential immunizing approach against murine schistosomiasis.

## 2. Materials and methods

### 2.1. Ethics statement

All experiments regarding animal housing and sacrifice have been approved by the Ethics Committee of Alexandria Faculty of Medicine based on Egyptian regulations for animal experimentation (Protocol approval number: 020732).

### 2.2. Vaccine preparation and characterization

*S*. *mansoni* cercariae were originally harvested from shedding *Biomphalaria alexandrina* snails infected with the Egyptian strain of *S*. *mansoni* purchased from the Schistosome Biologic Supply Centre, Theodor Bilharz Research Institute, Giza, Egypt. Snails were housed in glass aquaria containing snail-conditioned water and were fed lettuce leaves, tetramine fish food and calcium carbonate, under suitable environmental laboratory conditions. Maintenance of the *S*. *mansoni* life cycle was conducted between laboratory bred snails and Swiss strain Albino mice [25]. Cercariae of *S*. *mansoni* were gathered from infected snails by exposing them to light for 90 minutes, and were used for animal infection via paddling tail immersion method [26].

**Isolation of *S*. *mansoni* eggs** was done from livers of infected mice, 6–7 weeks post infection, using Smither՚s technique under complete aseptic conditions [27]. Freshly isolated eggs were thoroughly washed thrice with phosphate buffer saline (PBS) and maintained in Roswell Park Memorial Institute (RPMI) culture medium at 10^4^ egg/ml, containing 100 U of penicillin, 100 μg/ml of streptomycin and 0.25 μg/ml amphotericin B, at 37°C under 5% CO_2_ for 24 hours [23]. Following incubation, egg pellets were removed by centrifugation at 3000 Xg at 4°C for 20 minutes and the culture medium was collected for further EVs isolation.

**Isolation of EVs** was performed using ultracentrifugation technique, starting by centrifugation at 500 Xg followed by 2000 Xg and 4000 Xg for 20 min each. The supernatant was ultra-centrifuged at 120000 Xg for three hours (Beckmam Coulter, USA). All centrifugations’ steps were done at 4°C [22]. The final pellet containing EVs was resuspended in PBS and stored at -80°C for further use. **Characterization of EVs** was done using Lowry’s method for measuring the total amount of protein isolated [28], and transmission electron microscope to assess the size, shape and integrity of the isolated vesicles [20].

### 2.3. Animal groups and immunization protocol

The experiment was performed on 80 male Swiss strain Albino mice, four to six weeks old, 20–25 g each, that were free from parasites. Mice were provided from the animal house of the Medical Parasitology Department, Faculty of Medicine, Alexandria University. They were maintained under standard living conditions on a diet composed of wheat, bread, and milk on alternative days.

Mice were divided into 4 groups: Group I constituted 20 non-vaccinated control mice; which received three doses of PBS at two weeks intervals. Group II constituted adjuvanted control mice; that were injected by three doses of 100 μl alum. Group III constituted 20 vaccinated mice; which received three subcutaneous injections of EVs containing 20 μg protein (dose has been adjusted according to a pilot study). Group IV constituted 20 vaccinated adjuvanted mice; that were injected by three doses of EVs combined to alum.

All mice received their respective injections on days 1, 14, and 28 via subcutaneous route. Mice were infected two weeks after the last injection with 100 ± 10 cercariae, via paddling technique. They were sacrificed on day 84 from the start of experiment (six weeks post cercarial challenge) [26].

### 2.4. Vaccine evaluation

#### 2.4.1. Parasitological evaluation

**To estimate adult worm load,** infected mice were injected with 500-unit heparin then anaesthetized using overdose of thiopental sodium. Hepatic and porto-mesenteric vessels were perfused, and adult worms were recovered, counted to calculate couple and total adult count and the percent change was calculated [29].

**To calculate tissue egg count,** parts of the liver and intestine from infected mice were weighed, and each part was further cut into small pieces then was artificially digested by 10 ml of 4% potassium hydroxide for each gram of tissue. Eggs were counted per gram of tissue according to the method described by Cheever [29]. Pellegrino’s method was used to detect signs of maturity and immaturity of the eggs after their death [30].

**Ultrastructure changes** of all adults obtained from the four studied groups by perfusion technique were examined by transmission electron microscope (TEM) and scanning electron microscope (SEM), after preservation in 4% glutaraldehyde solution for TEM and 2.5% for SEM.

#### 2.4.2. Histopathological evaluation

Liver sections from all mice groups were fixed in 5% formalin for histopathological examination using haematoxylin and eosin (H&E) and Masson’s trichrome staining. Pathological changes were recorded, and the granulomata mean number and size were determined.

#### 2.4.3. Immunological evaluation

Blood samples were collected from the submandibular veins of all mice; two weeks after each injection and at the time of sacrifice. Blood samples were centrifuged at 8000 Xg for 10 min. Sera were stored at -20°C for further measuring of IgG and IFN γ levels using ELISA technique according to manufacturer’s instructions (Biospes co., Ltd, China) [31].

### 2.5. Detection of EVs components targeted by serum IgG antibodies [16]

Western immunoblotting was performed to identify EVs components that were targeted by serum antibodies. The protein marker used was of wide range MW; 3–198 (kDa). Mice sera obtained at the 84^th^ day from mice of infected control group I and experimental group III (infected, vaccinated) were used as the source of primary antibody. Electrotransfer of proteins from polyacrylamide gels to nitrocellulose membrane (Boster, USA) was done after electrophoresis at 60 volts. Fifteen μl of sample containing EVs was used and 6 μl of marker was loaded. Membrane was blocked using 5% of skimmed milk for one hour. Membrane was probed with serum (1:300 dilution in Tris-buffered saline with 0.5% V/V Tween 20 (TBST)). Membrane was incubated overnight in 4°C on a shaker. Bound antibody was detected by polyclonal goat anti mouse Ig/HRP (Dako Co., Denmark) in 1:1000 dilution in TBST. It was added in 5% skimmed milk in TBST and incubated for 1 hour. Membranes were washed in TBST between each of these steps. Enhanced Chemiluminescence mix was prepared and the membrane was incubated for 1–2 minutes. Results were visualized in a dark room. Gel documentation system (Geldoc-it, UVP, England), was applied for data analysis and more accurate determination of the band molecular weight using Total lab analysis software, (Ver.1.0.1).

### 2.6. Statistical analysis of the data

Data were analysed using IBM SPSS software package version 20.0. (Armonk, NY: IBM Corp). The Kolmogorov-Smirnov test was used to verify the normality of distribution. Quantitative data were described using range, mean and standard deviation. Significance of the obtained results was judged at the 5% level.

F-test (ANOVA) was used for normally distributed quantitative variables, to compare between more than two groups, and post-hoc test (Tukey) for pairwise comparisons. Kruskal Wallis test for abnormally distributed quantitative variables, to compare between more than two studied groups, and post-hoc (Dunn’s multiple comparisons test) for pairwise comparisons. Finally, ANOVA test was used with repeated measures, for normally distributed quantitative variables, to compare between more than two periods, and post-hoc test (Bonferroni adjusted) for pairwise comparisons. Percent change for different evaluation parameters was calculated according to the following formula:

%ch=(C−V)/C×100.

Where % ch is the precent of change, C is the mean of the control group and V is the mean of the vaccinated group.

## 3. Results

### 3.1. Characterization of EVs

**Fig 1:**
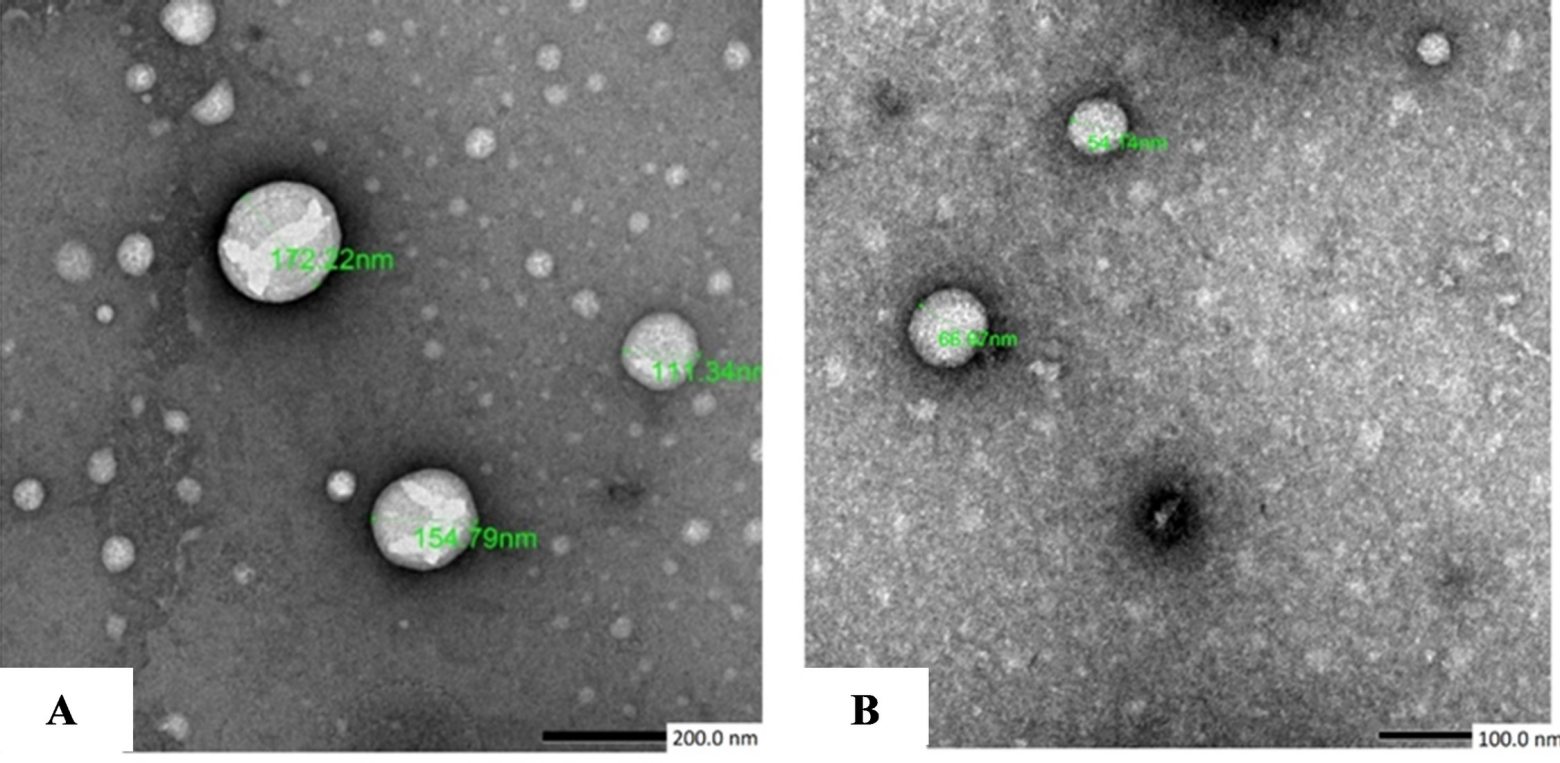
Transmission electron microscopic structure of the *Schistosoma mansoni*-egg derived extracellular vesicles (EVs) showing variable sized multiple EVs with regular rounded outline. (A: X 10000, B: X 15000).

As detected by TEM, EVs isolated from *S*. *mansoni* eggs exhibited a rounded morphology with uniform outline and variable sizes. Their sizes ranged from 50.5 nm to 172.2 nm (Fig 1A and 1B). Average amount of protein content obtained after ultracentrifugation of their culture supernatant, measured by Lowry’s technique was 345 μg/ml.

### 3.2. Vaccine evaluation

#### 3.2.1. Parasitological evaluation

**Fig 2:**
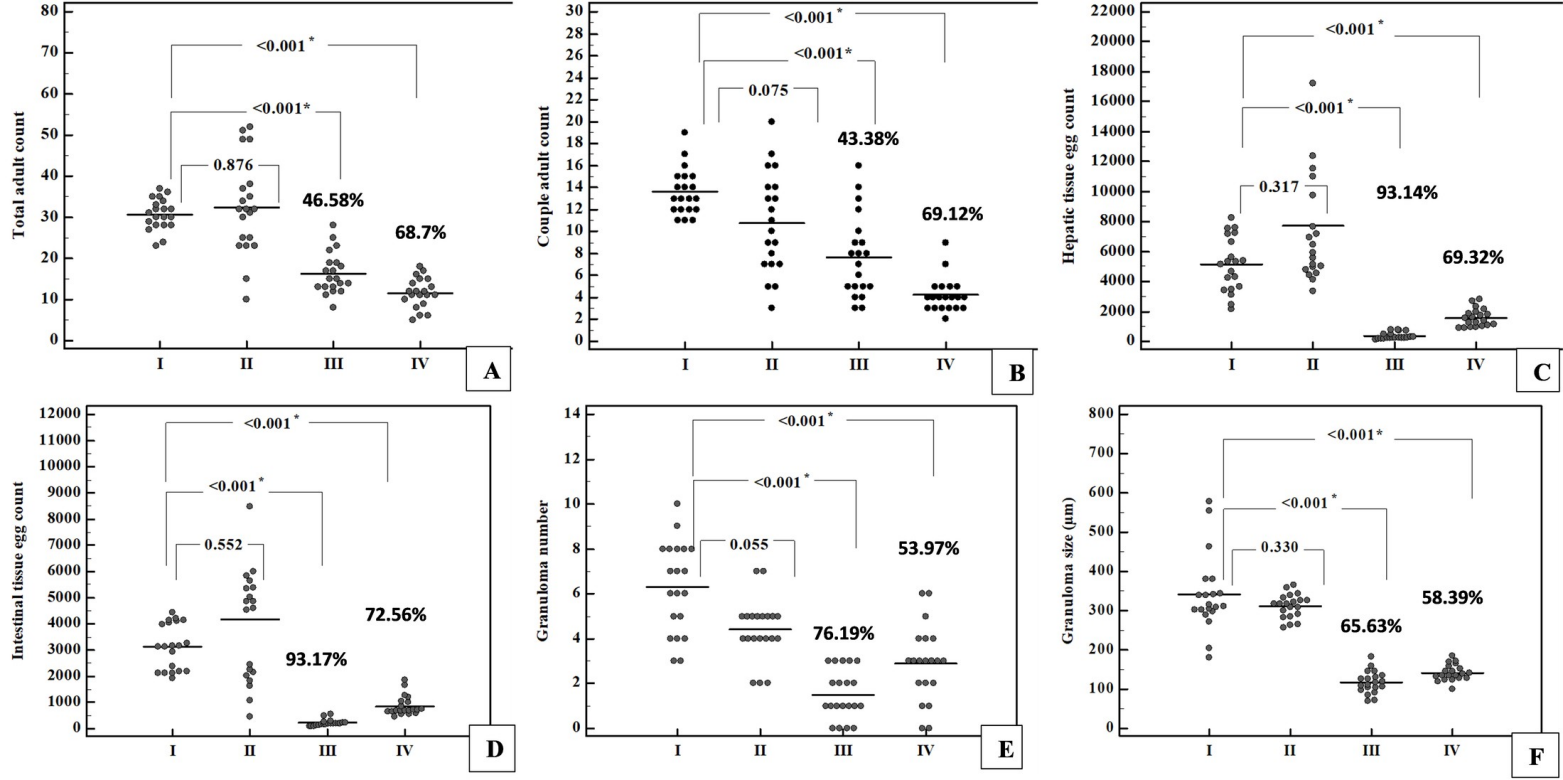
Scatter plot of parasitological evaluation parameters of the studied groups. **A**: Adult worm load in different studied groups obtained by perfusion technique 6 weeks post infection. **B**: Couple worm load in the studied groups **C**: Hepatic tissue egg count in the studied groups. **D**: Intestinal tissue egg count in the studied groups. **E**: Granuloma number in the livers of the studied groups. **F:** Granuloma size in micrometre measured in the livers of the studied groups. %: Represent the percent change. *: Statistically significant at p ≤ 0.05.

**Adult worm load estimation** showed that, mice vaccinated with EVs alone (group III) or in combination with alum (group IV) showed statistically significant reduction in their mean total adult worm load, with percent reduction 46.58% and 68.7% respectively (p ≤ 0.05) (Fig 2A). Also, the reduction was significant when comparing experimental groups (III & IV) to the infected adjuvanted control group (group II) (p ≤ 0.05). Statistical analysis showed non-significant difference between results of both experimental groups. Infected adjuvanted mice (group II) showed non-significant difference in adult count compared to infected control group (group I) (p > 0.05). While the couple count showed statistically significant reduction in both experimental groups (p ≤ 0.05) with percent reduction 43.38% in group III and 69.12% in group IV. On comparing results of both experimental groups, couple count in group IV showed statistically significant reduction (p ≤ 0.05) (Fig 2B and S1 Text).

**Tissue egg count** showed that both experimental groups (III &IV) had a statistically significant lower hepatic and intestinal egg counts compared to the infected control (group I) (p ≤ 0.05). The percent reduction for hepatic egg count of the infected vaccinated group III was 93.14% and 93.17% for intestinal egg count. While percent reduction of hepatic count in the infected vaccinated adjuvanted group IV was 69.32% and 72.56% for intestinal egg count, with statistical significance skewed to group III (p ≤ 0.05) (Fig 2C and 2D and S1 Text).

**Fig 3:**
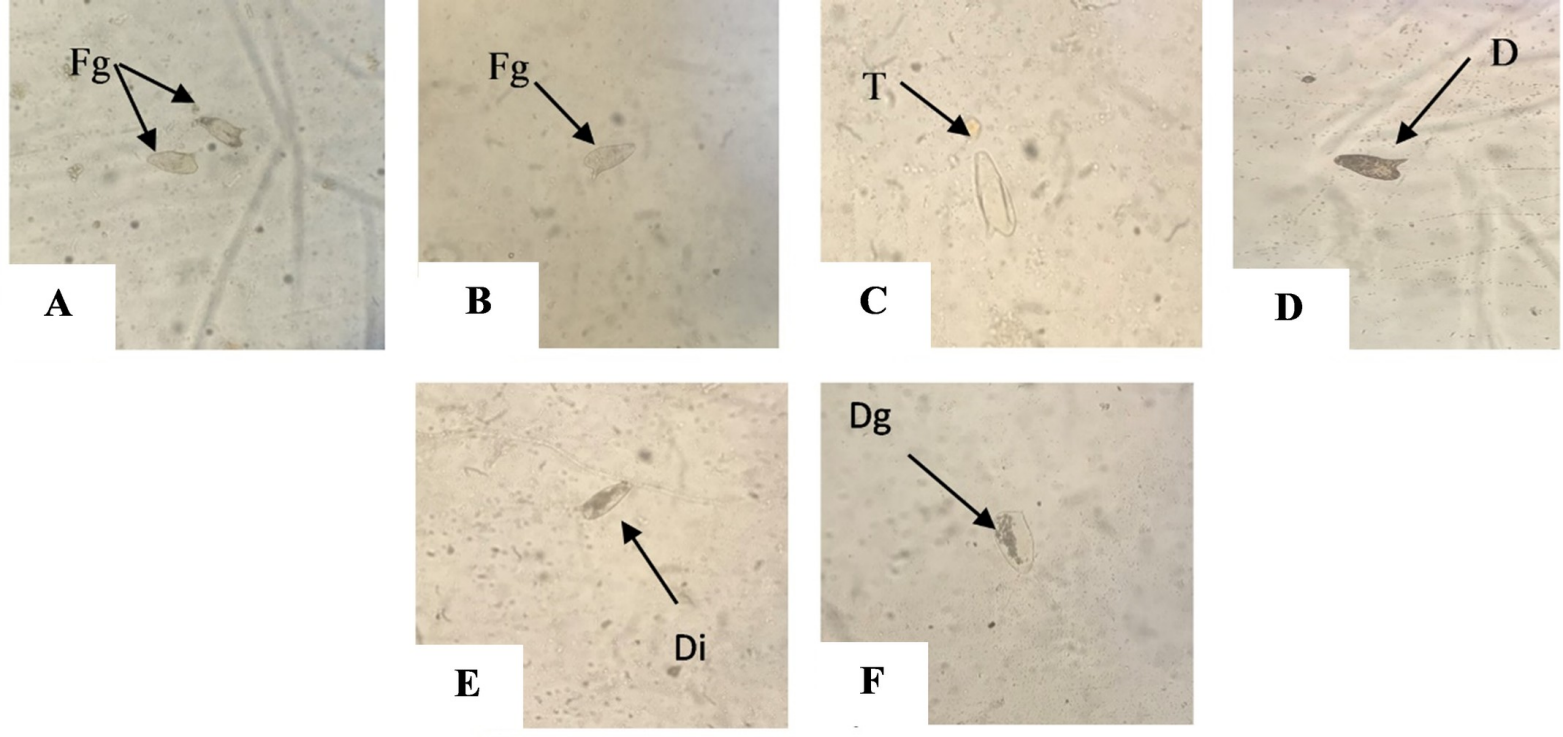
Morphological features of *S*. *mansoni* eggs retrieved after tissue digestion using Pellegrino’s method. (**A-D)** Eggs died immaturely. (**A &B)** Finely granular (Fg) eggs (X100). (**C)** Transparent (T) egg (X 100). (**D**) Darkened (D) egg (X 100). **(E-F)** Mature eggs. **(E)** Disintegrated (Di) egg (X 100). **(F)** Egg showing dense granules (Dg) (X 100).

Pellegrino’s method showed that eggs which died immature appeared with embryos at different stages of development. The wall of the embryo was well distinguished, and it occupied from one third up to more than two thirds of the longitudinal diameter of the egg. Some immature eggs appeared finely granular (Fig 3A and 3B), semi-transparent (Fig 3C), or darkened (Fig 3D). On the other hand, eggs that died after maturation either had disintegrating miracidium (Fig 3E), and others were roughly granular (Fig 3F).

By observing the morphology of 100 *S*. *mansoni* eggs from the infected control group, signs of immaturity were noted in 90% of the examined eggs, while 10% died post full maturation. However, eggs obtained from infected adjuvanted mice group II showed that 59% of eggs died after reaching maturity, versus 69% in the infected vaccinated group III. On the other hand, only 22% of eggs were immature in infected vaccinated and adjuvanted group IV.

**Fig 4:**
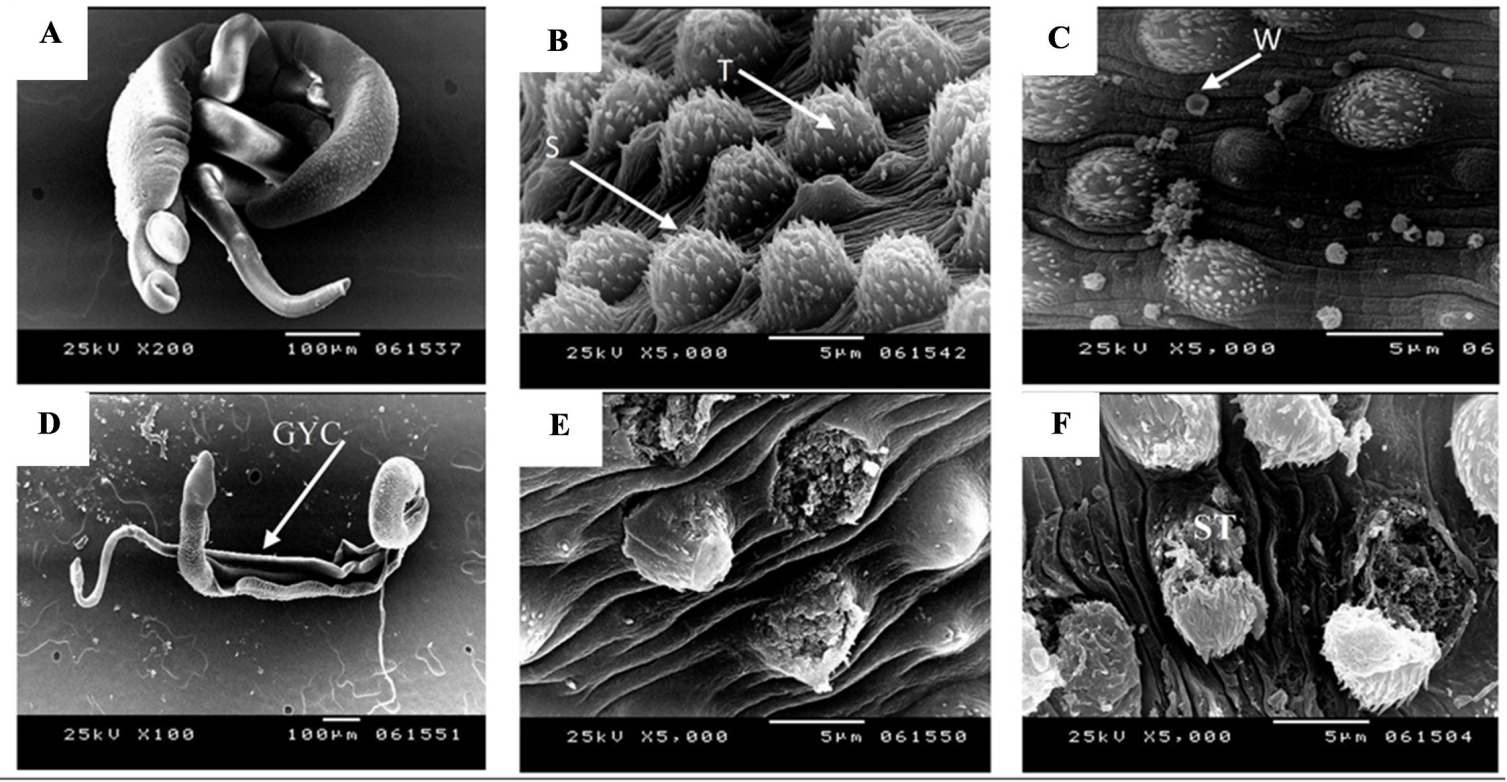
**Scanning electron micrographs of adult *S*. *mansoni* worms recovered from infected control group (A-B), infected vaccinated group (C-E) and infected vaccinated adjuvanted group (F). (A)** Adult couple worms obtained with normal configuration, muscle tone and normal male sucker (X 200). **(B)** Male dorsal tegument showing uniform size, distribution tubercles (T) and visible spines (S) (X 5000). **(C)** Dorsal tegument of adult male with white blood cell (W) attached to it (X 5000). **(D)** Distorted elongated couple with wide gynaecophoric canal (GYC) (X100). **(E)** Dorsal tegument with variably sized tubercles and complete loss of spines of male worm (X 5000). **(F)** Adult male worm with sloughing of the dorsal tegumental tubercles (ST) and lost spines (X 5000).

**Ultrastructural examination by SEM** of adult male, female and couple schistosomes in infected control groups (I & II) had normal morphological features with typical body configuration, typical gynaecophoric canal (Fig 4A) and tegumental structures (Fig 4B and 4C).

As regards infected vaccinated group III (Fig 4D and 4E), adult males and couples showed complete distortion of their tegument and loss of muscle tone with widening of gynaecophoric canal. The dorsolateral tegument was sloughed with flattening of the dorsal tubercles and shortened irregular spines. Altered dorsal tubercles revealed peeling and focal sloughing of their tegument, with occasional bursts of some tubercles and evident spine blunting. Swollen suckers were observed with loss of dorsal tubercles. Female adult worms showed a swollen area between the oral and ventral suckers with an evident dimple along with sucker’s oedema and blebbing. The posterior end was oedematous showing cauliflower appearance.

Adult male worms obtained from infected, vaccinated-adjuvanted group IV, showed the same ultrastructural changes as group III, with complete loss of tone, with distortion of the normal couple morphology and inability to maintain the female worm inside the gynaecophoric canal (Fig 4F).

**Fig 5:**
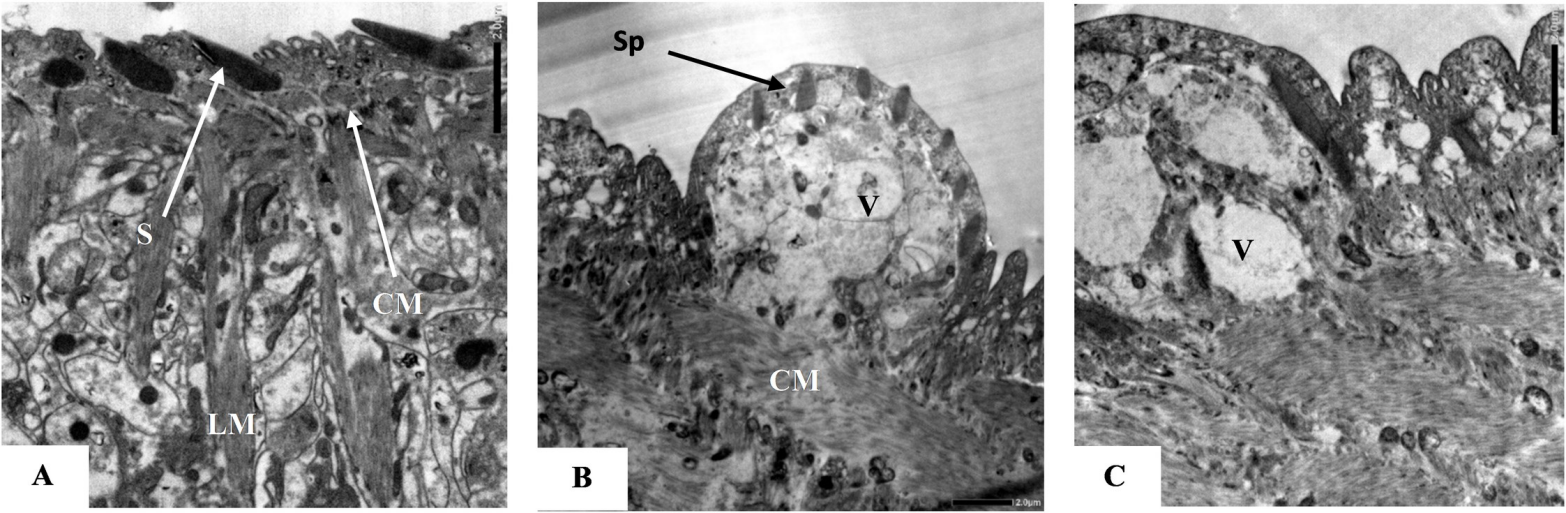
**TEM of adult male tegument in infected control group (A), infected vaccinated group (B) and infected vaccinated adjuvanted group (C). (A)** Normal outer tegumental layer showing large triangular spines (S), circular muscles (CM), and longitudinal muscles (LM) (X3000). **(B)** Blebbing of the tegument with deeply sunken spines (Sp), the subtegumental tissue shows vacuolation (V) with disruption of the circular muscles (Cm) (X 2000). **(C)** Irregularity of the tegument with absence of the spine, the subsegmental tissue shows vacuolation (V) with disruption of the circular muscle (X 2000).

**TEM results** of adult male worms obtained from infected control groups (I &II) showed normal tegumental spines. Both control groups showed normal appearance of the longitudinal and circular muscle layer (Fig 5A). As regards adult worms obtained from experimental groups III and IV, the tegumental layer showed swelling and blebbing in some areas with embedding or even total loss of the tegumental spines. The subtegumental layer showed evident vacuolation and swelling and disorganization of the muscles especially the circular muscle layer (Fig 5B and 5C).

#### 3.2.2. Histopathological results

**Fig 6:**
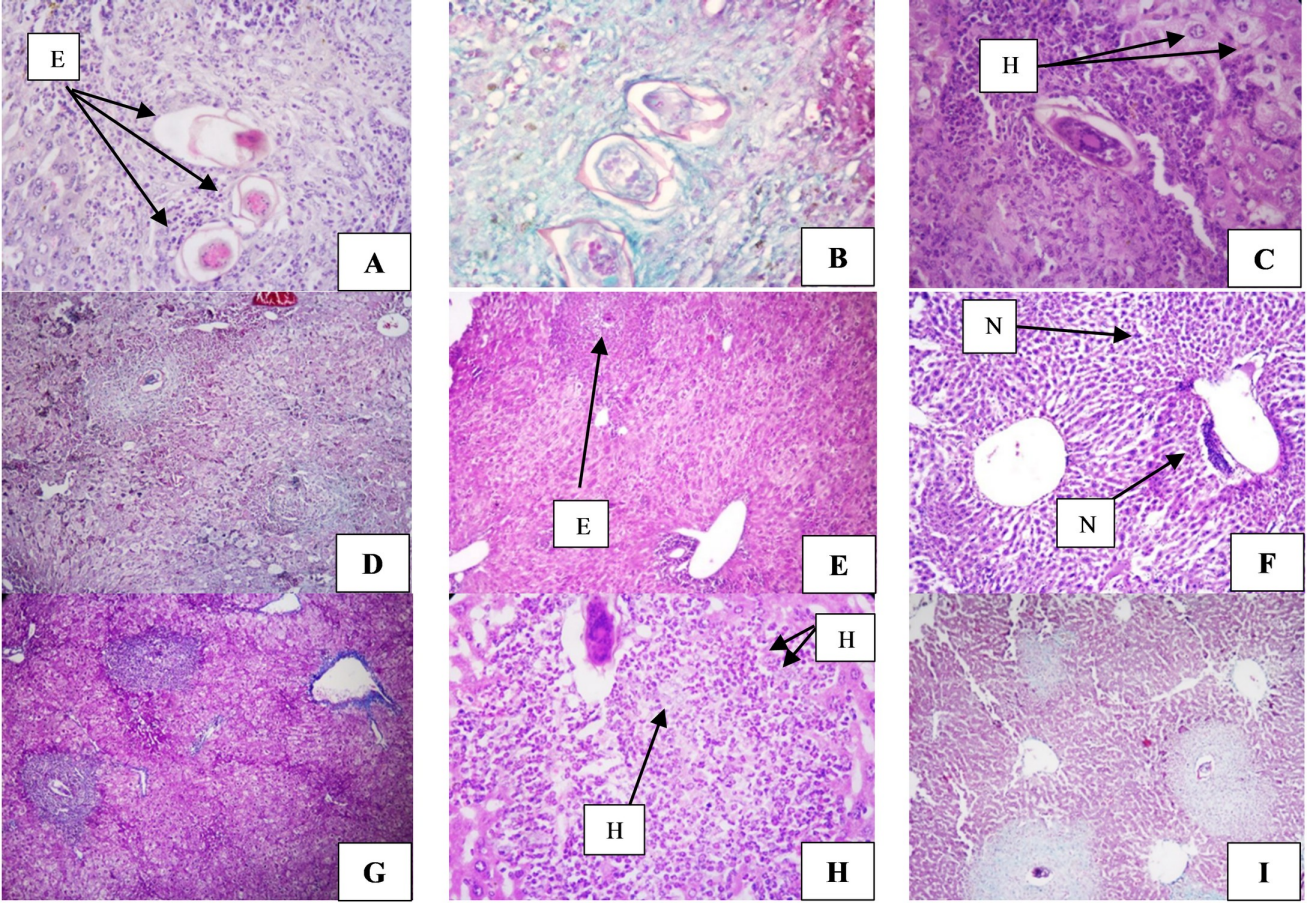
**Histopathological findings in liver sections of infected control group (A, B), infected adjuvanted control group (C, D), infected vaccinated group (E-G) and infected vaccinated adjuvanted group (H, I). (A)** H &E-stained section shows three eggs (E) surrounded by diffuse infiltrate (X400), **(B)** Trichrome-stained section showing fibrosis in granuloma around the eggs (X400). **(C)** H &E-stained section shows egg surrounded by granulomatous infiltrate formed mainly of histiocytes (H) (X400), **(D)** Trichrome-stained section showing fibrosis around the eggs. (X100). **(E)** H & E-stained liver section shows few eggs (E) surrounded by granulomatous reaction (X100), **(F)** H & E-stained liver section shows minimal infiltrate around the central vein with neutrophils infiltration (N) (X 400), **(G)** Trichrome -stained section showing fibrosis around the eggs with minimal fibrosis around central vein. **(H)** H & E-stained *S*. *mansoni* granulomatous rection formed mainly of histocytes (H) (X400), **(I)** Trichrome -stained section showing fibrosis around the eggs with minimal fibrosis around central vein (X100).

Liver sections from infected control group I showed numerous bilharzial ova surrounded by dense granulomata formed of large histiocytes, neutrophils, lymphocytes and eosinophils (Fig 6A). Masson’s trichrome stained sections showed severe fibrosis around the granulomata with moderate periportal and pericentral fibrosis (Fig 6B). While liver sections from infected adjuvanted mice (group II) showed eggs surrounded by granuloma formed mainly of histiocytes, with severe fibrosis around granulomata as seen by modified trichrome stain (Fig 6C and 6D).

Liver sections from infected vaccinated group III showed very few residual eggs surrounded by granulomatous reaction, moderate cellular infiltration was formed mainly of neutrophils with minimal infiltrate around the central vein (Fig 6E–6G). Vaccinated adjuvanted group IV showed few residual eggs, surrounded by moderate to severe tissue infiltrate formed mainly of histiocytes. Masson’s trichrome staining showed moderate fibrosis around the granulomata with minimal fibrosis around central vein in infected vaccinated mice (group III), while liver sections obtained from vaccinated adjuvanted mice showed severe fibrosis around the eggs with minimal fibrosis around portal and central vein (Fig 6H and 6I).

**Granuloma number and size estimation** showed that vaccination of mice with EVs with or without alum (group III & IV), resulted in significant reduction in both size and number of granuloma (p ≤ 0.05). The percent change was 76.19% for granuloma number and 65.63% for granuloma size in mice vaccinated with EVs alone (group III), combining EVs with alum (group IV) resulted in 53.97%, and 58.39% reduction in granuloma number and size respectively. It was observed that addition of alum to the vaccination regimen resulted in lesser reduction of both granuloma size and number, but this reduction was statistically non-significant regarding the granuloma number (p > 0.05) (Fig 2E and 2F and S1 Data).

#### 3.2.3. Immunological results

**Fig 7:**
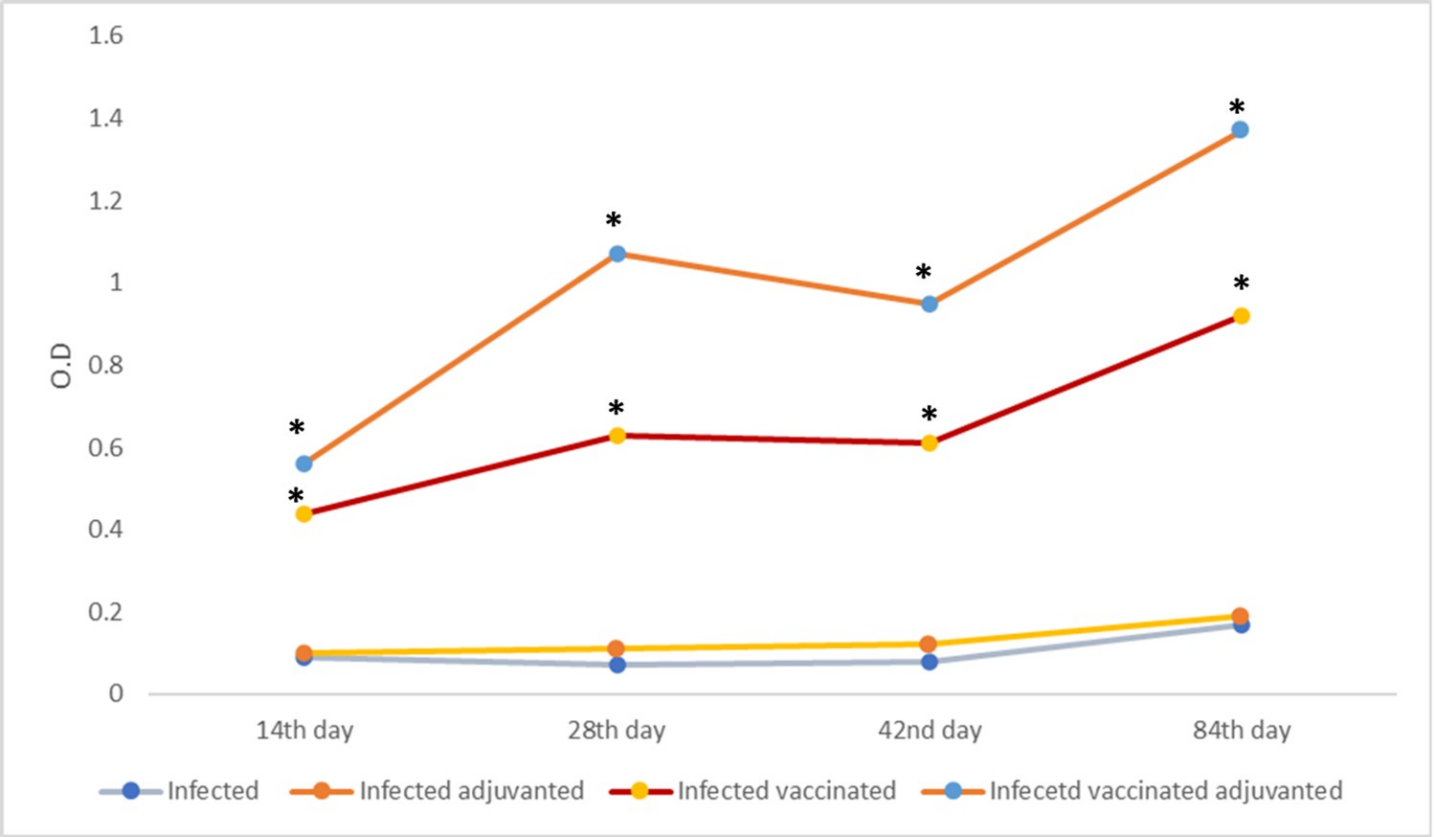
IgG profile in mice of different studied groups. Sera of mice were collected at days 14, 28, 42, and 84 after the first immunization and assayed by ELISA. Results are presented as the mean absorbance measured at 450 nm for each group. (*) Statistically significant at p ≤ 0.05.

**Antibody level (total IgG)** of control groups (group I and II) showed non-significant change in the IgG level along the study except six weeks post infection at the time of sacrifice (day 84) at which the antibody level significantly rose to 0.16 ± 0.03 O. D in group I and reaching 0.19 ± 0.04 O. D in group II compared to the previous reading (p ≤ 0.05) (Fig 7).

Mice vaccinated with EVs with or without alum (group III and IV) showed significant rise in the antibody level following the 1^st^ and 2^nd^ immunization doses followed by slight reduction in the mean IgG level after the 3^rd^ immunization dose, this reduction was significant only in mice vaccinated with EVs combined with alum. This reduction was followed by significant rise in both groups at the time of sacrifice reaching 0.92 ± 0.02 O. D in the vaccinated group III and 1.36 ± 0.11 O. D in infected vaccinated adjuvanted mice (group IV) (p ≤ 0.05). Alum combined with EVs resulted in significantly higher antibody level at all different time points of the study (p ≤ 0.05). The vaccine with or without adjuvant causes significantly higher IgG level compared to the infected control subgroups either with or without adjuvant (p ≤ 0.05).

**Fig 8:**
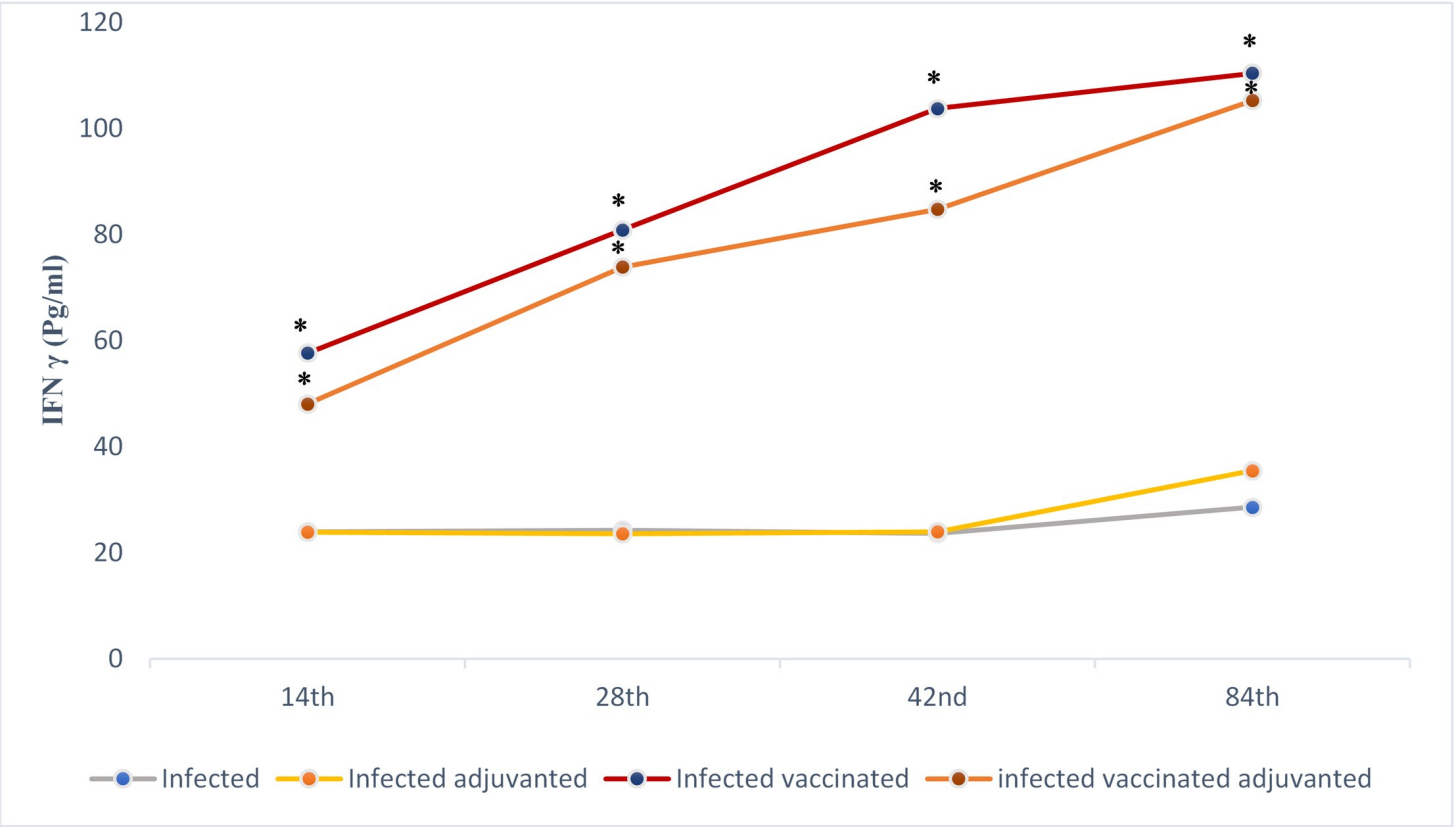
IFN γ levels in mice of different studied groups. Sera of mice were collected at days 14, 28, 42, and 84 after the first immunization and assayed by ELISA. (*) Statistically significant at p ≤ 0.05.

**On monitoring IFN γ level** in each group along different time of measurement, ELISA test for the sera obtained from infected control group I showed non-significant differences in the levels of IFN γ at each time point of the study except six weeks post infection (day 84) at which the level significantly rose to 28.6 ± 0.5 pg/ml. Infected adjuvanted mice (group II) did not show significant difference in IFN γ level along the study except at the time of sacrifice (p > 0.05). Alum alone did not cause any significant difference in IFN γ levels compared to the infected control group I (p > 0.05) (Fig 8).

Sera from mice vaccinated with EVs (with or without adjuvant) (group III & IV) showed continuous rise of IFN γ levels after each vaccination dose reaching 110.4 ±22.2 pg/ml at time of sacrifice of infected vaccinated group III and 105.3 ± 26.3 pg/ml in infected adjuvanted mice (group IV). Sera obtained from mice vaccinated with EVs alone (group III) showed statistically non-significant higher IFN γ level compared to those vaccinated with EVs combined with alum (group IV) (p > 0.05), except after the 3^rd^ immunization dose at which the rise was statistically significant (p ≤ 0.05). Vaccination with EVs (with or without alum) (group III and IV) results in statistically higher level of IFN γ compared to all control groups (I, & II) at different time of measurement (p ≤ 0.05).

### 3.3. Results of western blot test

**Fig 9:**
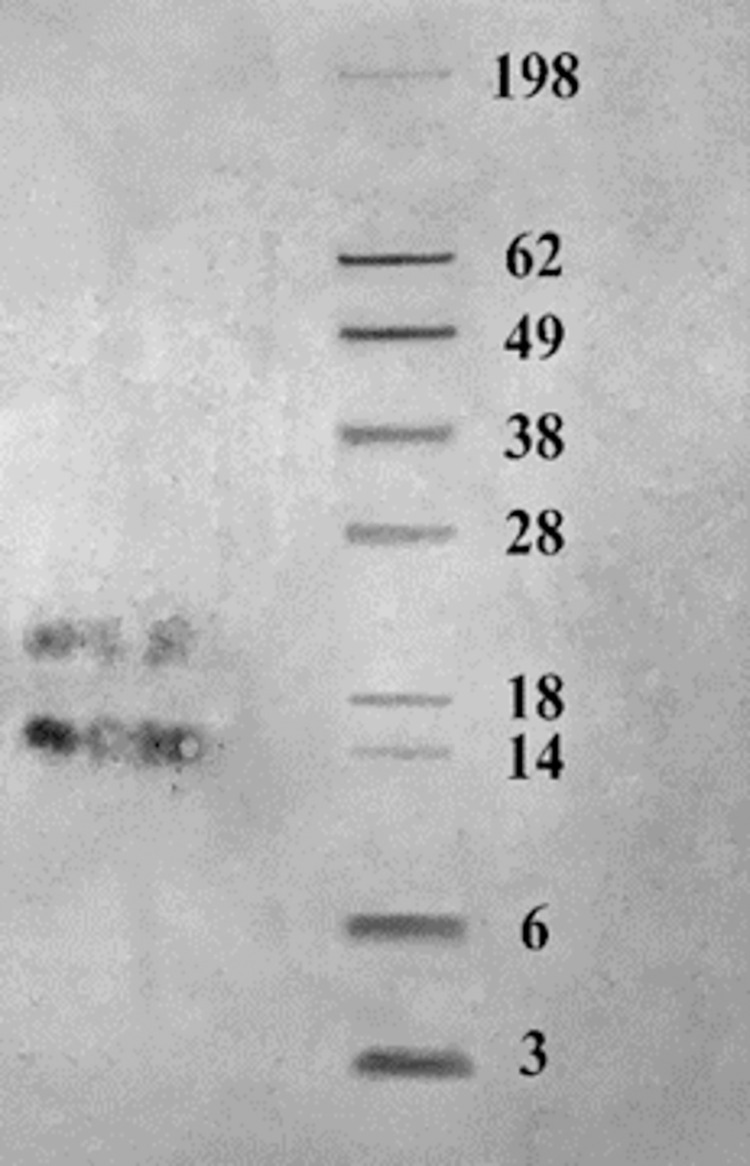
Two immunogenic bands obtained by western blot analysis of EVs using vaccinated mice sera as a source of primary antibody.

On performing the test using sera from the infected control group, no band was obtained. While using the sera from the experimental group III resulted in two bands. The first band was at ~21 KDa while the second band was opposite to the marker band of 14 KDa (Fig 9). Molecular weight of the bands was confirmed by gel documentation system analysis.

## 4. Discussion

The egg stage is a determinant turning point in *S*. *mansoni* infection [2]. Its bioactive products play a central role in evoking strong immune response [2]. For the first time, *S*. *mansoni* egg-derived EVs’ immune protective role was evaluated against experimental schistosomiasis mansoni.

In the current study, identification of EVs isolated from *S*. *mansoni* eggs by TEM showed uniform sized and shaped vesicles; thus ruling out the possibility of apoptotic and necrotic bodies [32]. Their size range was comparable to those obtained from *S*. *mansoni* adult and *S*. *japonicum* with slight difference [22,23]. This diversity of the size could be explained by the difference in the sources of EVs [32].

In the current study, both experimental groups showed statistically significant changes in the different evaluation parameters, compared to their control groups (p ≤ 0.05). In experimental group III, vaccination with *S*. *mansoni* egg-derived EVs induced a significant reduction rate in the total adult worm loads. Using EVs in immunization against parasitic infections showed variable reduction rates in the adult worm load. EVs isolated from *Opisthorchis viverrini* showed 27% reduction [15], while those of *Heligmosomoides polygyrus* resulted in 82% reduction in adult count [14]. *Echinostoma*-derived EVs did not result in reduction in the adult worm count despite inducing improvement of the clinical outcome of the infection [17].

Worm load reduction rate in the current study is comparable to that obtained by using *S*. *mansoni* rP22 caused 51% adult worm reduction [33], also using rSm14 as immunizing agent (67%) [34]. While using TSP-2 recombinant protein resulted only in 34% reduction of the total worm load [35]. When *S*. *mansoni* infected mice were immunized by TSP-2 protein derived from *S*. *haematobium* adult EVs, it resulted in 22% adult worm reduction [21]. Using egg secretory/excretory products; Kunitz-type protease inhibitor (KI-1) resulted in partial protection with 23–33% adult load reduction [36].

Reduction in the adult count in the present study could be explained through evident tegumental derangements as detected by SEM in the experimental groups. This exposure of the underlying antigenic determinants to the host immune cells could have led to enhanced vaccine protective efficacy [37], as white blood cells (WBCs) were demonstrating attacking the tegument. These ultrastructural changes simulate these of El-Shabasy et al (2015), who studied tegumental changes caused by the radiation-attenuated cercarial vaccine [38]. Schistosomal tegument is considered an important vaccine target for its role in parasite immune evasion and other biological functions including parasite nutrition, osmoregulation and signal transduction. Tegument proteins of the parasite are vulnerable to the attack by the host immune system [39].

TEM revealed that male’s tegumental derangement was associated with loss of subtegumental muscle tone which is mostly the underlying cause for the observed evident widening of the gynaecophoric canal in experimental mice. This could have probably hindered the appropriate coupling of males with females. The muscular action of the clasping male helps females to get blood nutrition from the host and is essential for oocyte maturation as well [40]. Decoupling could explain the reduction of adult worms not merely on the basis of vaccine effects on the female worms, but also due to the vulnerable role of adult males in females’ survival, maintenance and fecundity [40], which in turn, could have impacted the tissue egg count.

The strong immune response directed toward the adult worm, represented as extensive tegumental damage, could be explained by the presence of antigens from different developmental stages of the parasite such as the adult stage carried by the EVs [13,20]. An example of these proteins is TSPs, which are considered as one of the molecular markers of EVs [21]. They are present on the surface membrane of EVs derived from different parasites [41]. TSPs have important roles in EVs biogenesis and adult schistosomal tegumental development. Studies showed that production of antibodies against EVs-derived TSP played an important role in protection against different parasitic infections; including *Schistosoma* [15,18,19].

Significant egg count and granuloma size reduction in group III could be linked to the reduction of the adult worm load in addition to the decoupling effect. Du et al (2020) showed that cargo of *S*. *japonicum* EVs plays an important role in egg production [42]. Inhibition of this role may be an additional factor that led to reduction in the egg count.

It was observed that the egg count was significantly reduced in group III in comparison to group IV, although the adult counting showed higher reduction rate in group IV in both couple and total count. This could be explained by the fact that Th2 environment in the prepatent period of schistosomiasis is important for female worm maturation and reproduction [43,44]. In the current work IFN γ level was higher in group III indicating more skewing of the immune response toward Th1 environment, compromising the female worm maturation, fecundity and reproduction.

Egg reduction is more influential in vaccine efficacy than reduction of the adult worms, as hepatic egg count reduction reflects diminution in egg entrapped in liver leading to improvement in liver pathology, in addition to intestinal egg count reduction, that leads to decrease the pathology as well as transmission rate of the parasite [36].

As regards eggs maturity, reduction of the immature eggs percentage in experimental groups as shown by Pellegrino’s method, goes in parallel with the significant reduction in the adult female count recorded in the present study. These changes could be explained by the cessation of oviposition due to the death of adult worms, or reduction of female fecundity due to decoupling effect [45]. These results reflect that the reduction of the granuloma number could be related to the reduction of the tissue egg count rather than to the inability of the existing eggs to stimulate granuloma formation.

Using EVs from various parasites as immunizing agent resulted in significant reduction in egg count reaching 22.5%-32% [14,15,17]. Rezende et al (2011) proved that immunization with schistosomal rP22 resulted in 22.5% reduction in hepatic egg count [33]. Likewise, TSP-2 as an immunizing agent resulted in 52% reduction in hepatic egg count [35]. Egg’s excretory/secretory product resulted in only 39% reduction of the hepatic egg count, while SEA resulted in 70.87% reduction [36,46].

In the current study, parasitological changes were accompanied by strong immune response represented by continuous rise in the level of IFN γ and IgG in both experimental groups. Being nano-sized vesicles, EVs are easily taken up by the immune cells. Subsequently, proteins contained inside them are better engulfed by the antigen presenting cell, thus increasing their antigenicity [16].

The gradual rise of IFN γ profile in group III mice, vaccinated with EVs compared to infected control group can be explained in light of the results of Paul et al (2020), who verified that, EVs cargo internalised by mouse CD4^+^ T cells causes down modulation of Th2 cell differentiation and function, with respective flourishing of Th1 response resulting in IFN γ increment [47]. This function was demonstrated to be mediated by miRNA enclosed in the schistosomal EVs, which are a group of small non coding RNAs [48]. This miRNA down regulates Th cells important transcriptional program leading to decreased production of the Th2 cytokine [24]. It has been documented that treating macrophages (M) with *S*. *japonicum-*derived EVs led to its polarization toward M1, with subsequent increase in pro-inflammatory cytokines [49]. This has been proven to be mediated by EVs containing miRNA [48].

The sustained gradual rise in IFN γ could be explained by the fact that encapsulating antigens in lipid nano-spheres as EVs, protects them from degradation and enables slow release of antigen over time [16,50].

This high IFN γ level could explain the reduction of fibrosis around the portal tract and central vein, besides amelioration of hepatic inflammatory reaction and the high diminution in granuloma size (65.63%). Studies relate the reduction in the granuloma size and fibrosis to the switch in the normal Th2 response to Th1 response with the consequent increment in type 1 cytokines; including IFN γ [51]. This goes with Kaplan et al (1998) and Jankovic et al (1999), who demonstrated that decreased fibrosis in the murine model related to diminished production of type 2 and increased production of type 1 cytokines, rather than diminution of the worm or tissue egg burdens [52,53]. Transferred schistosomal EVs miRNA has been proven to attenuate liver fibrosis and hepatic pathological progression [23]. Also, lack of M2 production induced by EVs cargo might have a role in granuloma size reduction [54].

In the current study, in group III mice, vaccinated with EVs, neutrophils were the dominant cell in their hepatic granuloma. As known, neutrophils are recruited and accumulated at the periphery of the granuloma, releasing a number of proteins involved in collagen degradation and reabsorption [55].

Furthermore, vaccination of group III mice with EVs led to high IgG level compared to the infected control group. Antibodies appeared two weeks after the first immunization and continued to rise after the second immunization dose (day 28), with slight reduction after the third immunization dose (day 42). This pattern of IgG elevation has been previously recorded by Fonseca et al (2004) upon using rSm14 antigen alone or combined to alum as an immunizing agent [56].

Antibodies are involved in different mechanisms of parasite elimination. Antibody-dependent cell mediated cytotoxicity (ADCC) is involved in schistosomal killing and elimination [57]. Neutralizing antibody directed against EVs proteins block parasite EVs internalization to the host cells, which is an important mechanism in establishing parasitic infection [58]. Moreover, antibodies against schistosomal antigens are involved in the reduction of female fecundity [59].

Chaiyadet et al (2019) suggested that the immune protective effect induced by antibodies provoked by *Opisthorchis viverrini-*derived EVs could be due to blockage of EVs functions, by hindering their entry to host cells [15]. Antibody mediated blockage of entry *S*. *japonicum* EVs cargo into the hepatocyte results in reduction of hepatic fibrosis [13].

On the other hand, in the present study, immunizing mice of group IV with EVs in combination with alum apparently resulted in the less polarization of the immune response towards Th1 response, as proved by lesser production of IFN γ compared to the vaccinated group (III). This is caused by the documented Th2 stimulation properties of alum [60]. This adjuvant activates the humoral defence mechanisms by activating Th follicular cells important to memory B cells and plasma cells differentiation [60]. This could explain the higher IgG level compared to group III. Mostly, it could be presumed that lower IFN γ level led to the lesser reduction in granuloma size and the more fibrotic granuloma as compared to group III. Histiocytes were the dominant granulomatous cell type; which are known to facilitate collagen synthesis, contributing to the fibrosis-related pathology induced by schistosomiasis [61]. While the higher IgG level induced by combining EVs with alum is mostly the cause of more reduction in the adult count, as adult worms are killed mainly by ADCC mechanism [57]. These results go with the ‘happy valley hypothesis’ of Wilson et al (1999) who stated that in natural infection, the parasite appears most comfortable in Th0 which is an intermediate zone between Th1 and Th2 with no dominant immune response, and is represented by the ‘valley floor’. So, polarization to either Th1 or Th2 immune arms by vaccination process could be protective [62]. In the current study, being a Th2 stimulant, alum resulted in slight disturbance in the Th1 polarization caused by the EVs, causing slight reduction in the vaccine efficacy compared to group III. Results of the present study are comparable with that of Mossallam et al (2014), who showed that reduction in egg count and granuloma size was more in mice vaccinated with the antigen alone than mice vaccinated with antigen combined with alum, while adult reduction was more in mice vaccinated with antigen combined to alum [37].

On performing western immunoblotting, using sera from EVs-vaccinated group (III) resulted in recognition of two bands approximate molecular weights 14 and 21 KDa. This suggests that one of the protective mechanisms of the EVs is the ability to induce specific antibody response against its proteins. This result goes with the results of Shears et al (2018), who confirmed that *Trichuris* derived EVs are able to induce protective antibody response upon using it as immunizing agent against *Trichuris* infection [16].

In conclusion, the present study highlights the immune protective role of *S*. *mansoni* egg-derived EVs as a potential vaccine candidate against murine schistosomiasis mansoni. Significant increment of IFN γ and IgG profiles proves that egg derived EVs are able to modulate the immune system. Serum IgG strongly recognises two protein bands of ~14 KDa and ~ 21 KDa. Vaccine under study is able to achieve 46.58% reduction of tegumentally-deranged adult worms. Hepatic and intestinal egg counts reduction were 93.14–93.17%, accompanied by 65.63% reduction of hepatic granulomata sizes, with remarkable amelioration of their histopathology. Whereas, adjuvanted EVs did not significantly influence the protection any further. Proteomic analysis is currently planned for precise confirmation of the identity of immunogenic proteins involved in eliciting the immune response. Also, future trials of different adjuvants may result in higher protection rate.

## Supporting information

S1 Text Statistical analysis of results of adult worm load, egg load and granuloma size and number six weeks post infection. (DOCX)

S1 Data Results of the studied groups as regards parasitological and histopathological parameter. (XLSX)

## References

[1] Nelwan ML. Schistosomiasis: Life Cycle, Diagnosis, and Control. Curr Ther Res Clin Exp. 2019;9:5–9. doi: 10.1016/j.curtheres.2019.06.001 . 31372189 PMC6658823

[2] Costain A, MacDonald A, Smits HH. Schistosome Egg Migration: Mechanisms, Pathogenesis and Host Immune Responses. Front Immunol. 2018;9:3042. doi: 10.3389/fimmu.2018.03042 . 30619372 PMC6306409

[3] Shaker Y, Samy N, Ashour E. Hepatobiliary Schistosomiasis. JCTH. 2014;2(3):212–6. Epub 09/15. doi: 10.14218/JCTH.2014.00018 . 26357627 PMC4521248

[4] Abou-El-Naga IF, Amer EI, Boulos LM, El-Faham MH, Abou Seada NM, Younis SS. Biological and proteomic studies of *Schistosoma mansoni* with decreased sensitivity to praziquantel. Comp Immunol Microbiol Infect Dis. 2019;66:101341. Epub 2019/08/23. doi: 10.1016/j.cimid.2019.101341 . 31437686

[5] Abou-El-Naga IF. Towards elimination of schistosomiasis after 5000 years of endemicity in Egypt. Acta Trop. 2018;181:112–21. doi: 10.1016/j.actatropica.2018.02.005 29453950

[6] Verani JR, Abudho B, Montgomery SP, Mwinzi PN, Shane HL, Butler SE, et al. Schistosomiasis among young children in Usoma, Kenya. Am J Trop Med Hyg. 2011;84(5):787–91. doi: 10.4269/ajtmh.2011.10-0685 21540390 PMC3083748

[7] Kelly EA, Colley DG. In vivo effects of monoclonal anti-L3T4 antibody on immune responsiveness of mice infected with *Schistosoma manson*i. Reduction of irradiated cercariae-induced resistance. J Immunol. 1988;140(8):2737–45. Epub 1988/04/15. . 3128606

[8] McManus DP, Loukas A. Current status of vaccines for schistosomiasis. Clin Microbiol Rev. 2008;21(1):225–42. Epub 2008/01/19. doi: 10.1128/CMR.00046-07 ; PubMed Central PMCID: PMC2223839. 18202444 PMC2223839

[9] Tendler M, Almeida MS, Vilar MM, Pinto PM, Limaverde-Sousa G. Current Status of the Sm14/GLA-SE Schistosomiasis Vaccine: Overcoming Barriers and Paradigms towards the First Anti-Parasitic Human(itarian) Vaccine. Trop Med Infect Dis. 2018;3(4):121. Epub 2018/11/25. doi: 10.3390/tropicalmed3040121 ; PubMed Central PMCID: PMC6306874. 30469320 PMC6306874

[10] Riveau G, Schacht A-M, Dompnier J-P, Deplanque D, Seck M, Waucquier N, et al. Safety and efficacy of the rSh28GST urinary schistosomiasis vaccine: A phase 3 randomized, controlled trial in Senegalese children. PLoS Negl Trop Dis. 2018;12(12):e0006968–e. doi: 10.1371/journal.pntd.0006968 . 30532268 PMC6300301

[11] Keitel WA, Potter GE, Diemert D, Bethony J, El Sahly HM, Kennedy JK, et al. A phase 1 study of the safety, reactogenicity, and immunogenicity of a *Schistosoma mansoni* vaccine with or without glucopyranosyl lipid A aqueous formulation (GLA-AF) in healthy adults from a non-endemic area. Vaccine. 2019;37(43):6500–9. Epub 2019/09/14. doi: 10.1016/j.vaccine.2019.08.075 ; PubMed Central PMCID: PMC6771426. 31515141 PMC6771426

[12] Raposo G, Stoorvogel W. Extracellular vesicles: exosomes, microvesicles, and friends. J Cell Biol. 2013;200(4):373–83. Epub 2013/02/20. doi: 10.1083/jcb.201211138 ; PubMed Central PMCID: PMC3575529. 23420871 PMC3575529

[13] Zhu L, Liu J, Dao J, Lu K, Li H, Gu H, et al. Molecular characterization of *S. japonicum* exosome-like vesicles reveals their regulatory roles in parasite-host interactions. Sci Rep. 2016;6:25885. Epub 2016/05/14. doi: 10.1038/srep25885 ; PubMed Central PMCID: PMC4865838. 27172881 PMC4865838

[14] Coakley G, McCaskill JL, Borger JG, Simbari F, Robertson E, Millar M, et al. Extracellular Vesicles from a Helminth Parasite Suppress Macrophage Activation and Constitute an Effective Vaccine for Protective Immunity. Cell Rep. 2017;19(8):1545–57. doi: 10.1016/j.celrep.2017.05.001 28538175 PMC5457486

[15] Chaiyadet S, Sotillo J, Krueajampa W, Thongsen S, Brindley PJ, Sripa B, et al. Vaccination of hamsters with *Opisthorchis viverrini* extracellular vesicles and vesicle-derived recombinant tetraspanins induces antibodies that block vesicle uptake by cholangiocytes and reduce parasite burden after challenge infection. PLoS Negl Trop Dis. 2019;13(5):e0007450. doi: 10.1371/journal.pntd.0007450 31136572 PMC6555531

[16] Shears R, Bancroft A, Hughes G, Grencis R, Thornton D. Extracellular vesicles induce protective immunity against *Trichuris muris*. Parasite Immunol. 2018;40(7):e12536. Epub 2018/05/11. doi: 10.1111/pim.12536 ; PubMed Central PMCID: PMC6055854. 29746004 PMC6055854

[17] Trelis M, Galiano A, Bolado A, Toledo R, Marcilla A, Bernal D. Subcutaneous injection of exosomes reduces symptom severity and mortality induced by *Echinostoma caproni* infection in BALB/c mice. Int J Parasitol. 2016;46(12):799–808. Epub 2016/10/25. doi: 10.1016/j.ijpara.2016.07.003 . 27590846

[18] Kifle DW, Chaiyadet S, Waardenberg AJ, Wise I, Cooper M, Becker L, et al. Uptake of *Schistosoma mansoni* extracellular vesicles by human endothelial and monocytic cell lines and impact on vascular endothelial cell gene expression. Int J Parasitol. 2020;50(9):685–96. doi: 10.1016/j.ijpara.2020.05.005 32598872

[19] Chaiyadet S, Sotillo J, Smout M, Cantacessi C, Jones MK, Johnson MS, et al. Carcinogenic Liver Fluke Secretes Extracellular Vesicles That Promote Cholangiocytes to Adopt a Tumorigenic Phenotype. J Infect Dis. 2015;212(10):1636–45. Epub 2015/05/20. doi: 10.1093/infdis/jiv291 ; PubMed Central PMCID: PMC4621255. 25985904 PMC4621255

[20] Nowacki FC, Swain MT, Klychnikov OI, Niazi U, Ivens A, Quintana JF, et al. Protein and small non-coding RNA-enriched extracellular vesicles are released by the pathogenic blood fluke *Schistosoma mansoni*. J Extracell Vesicles. 2015;4:28665. Epub 2015/10/08. doi: 10.3402/jev.v4.28665 ; PubMed Central PMCID: PMC4595467. 26443722 PMC4595467

[21] Mekonnen GG, Tedla BA, Pickering D, Becker L, Wang L, Zhan B, et al. *Schistosoma haematobium* Extracellular Vesicle Proteins Confer Protection in a Heterologous Model of Schistosomiasis. Vaccines. 2020;8(3):416. Epub 2020/07/30. doi: 10.3390/vaccines8030416 ; PubMed Central PMCID: PMC7563238. 32722279 PMC7563238

[22] Sotillo J, Pearson M, Potriquet J, Becker L, Pickering D, Mulvenna J, et al. Extracellular vesicles secreted by *Schistosoma mansoni* contain protein vaccine candidates. Int J Parasitol. 2016;46(1):1–5. Epub 2015/10/16. doi: 10.1016/j.ijpara.2015.09.002 . 26460238

[23] Zhu S, Wang S, Lin Y, Jiang P, Cui X, Wang X, et al. Release of extracellular vesicles containing small RNAs from the eggs of *Schistosoma japonicum*. Parasit vectors. 2016;9(1):574. doi: 10.1186/s13071-016-1845-2 . 27825390 PMC5101684

[24] Meningher T, Barsheshet Y, Ofir-Birin Y, Gold D, Brant B, Dekel E, et al. Schistosomal extracellular vesicle-enclosed miRNAs modulate host T helper cell differentiation. EMBO reports. 2020;21(1):e47882–e. Epub 12/11. doi: 10.15252/embr.201947882 . 31825165 PMC6944914

[25] Abou-El-Naga IF, Sadaka HA, Amer EI, Diab IH, Khedr SI. Impact of the age of *Biomphalaria alexandrina* snails on *Schistosoma mansoni* transmission: modulation of the genetic outcome and the internal defence system of the snail. Mem. Inst Oswaldo Cruz. 2015;110(5):585–95. Epub 2015/06/11. doi: 10.1590/0074-02760150016 ; PubMed Central PMCID: PMC4569820. 26061235 PMC4569820

[26] Olivier L, Stirewalt MA. An efficient method for exposure of mice to cercariae of *Schistosoma mansoni*. J Parasitol. 1952;38(1):19–23. Epub 1952/02/01. . 14928147

[27] Smithers SR. The isolation of viable schistosome eggs by a digestion technique. Trans R Soc Trop Med Hyg. 1960;54:68–70. Epub 1960/01/01. doi: 10.1016/0035-9203(60)90214-5 . 13832187

[28] Peterson GL. A simplification of the protein assay method of Lowry et al. which is more generally applicable. Anal Biochem. 1977;83(2):346–56. Epub 1977/12/01. doi: 10.1016/0003-2697(77)90043-4 . 603028

[29] Cheever AW. Conditions affecting the accuracy of potassium hydroxide digestion techniques for counting *Schistosoma mansoni* eggs in tissues. Bull World Health Organ. 1968;39(2):328–31. . 4881073 PMC2554554

[30] Pellegrino J, Oliveira CA, Faria J, Cunha AS. New approach to the screening of drugs in experimental schistosomiasis mansoni in mice. Am J Trop Med Hyg. 1962;11:201–15. doi: 10.4269/ajtmh.1962.11.201 14484966

[31] Cardoso FC, Macedo GC, Gava E, Kitten GT, Mati VL, de Melo AL, et al. *Schistosoma mansoni* tegument protein Sm29 is able to induce a Th1-type of immune response and protection against parasite infection. PLoS Negl Trop Dis. 2008;2(10):e308–e. doi: 10.1371/journal.pntd.0000308 . 18827884 PMC2553283

[32] Wu Y, Deng W, Klinke DJ 2nd. Exosomes: improved methods to characterize their morphology, RNA content, and surface protein biomarkers. Analyst. 2015;140(19):6631–42. doi: 10.1039/c5an00688k . 26332016 PMC4986832

[33] Rezende CM, Silva MR, Santos IG, Silva GA, Gomes DA, Goes AM. Immunization with rP22 induces protective immunity against *Schistosoma mansoni*: effects on granuloma down-modulation and cytokine production. Immunol Lett. 2011;141(1):123–33. Epub 2011/09/29. doi: 10.1016/j.imlet.2011.09.003 . 21945176

[34] Tendler M, Brito CA, Vilar MM, Serra-Freire N, Diogo CM, Almeida MS, et al. A *Schistosoma mansoni* fatty acid-binding protein, Sm14, is the potential basis of a dual-purpose anti-helminth vaccine. Proc Natl Acad Sci USA. 1996;93(1):269–73. doi: 10.1073/pnas.93.1.269 . 8552619 PMC40220

[35] Eyayu T, Zeleke AJ, Worku L. Current status and future prospects of protein vaccine candidates against *Schistosoma manson*i infection. Parasite Epidemiol Control. 2020;11:e00176–e. doi: 10.1016/j.parepi.2020.e00176 . 32923703 PMC7475110

[36] Ranasinghe SL, Duke M, Harvie M, McManus DP. Kunitz-type protease inhibitor as a vaccine candidate against schistosomiasis mansoni. Int J Infect Dis. 2018;66:26–32. Epub 2017/11/13. doi: 10.1016/j.ijid.2017.10.024 . 29128645

[37] Mossallam SF, Amer EI, Ewaisha RE, Khalil AM, Aboushleib HM, Bahey-El-Din M. Fusion protein comprised of the two *schistosomal antigens*, Sm14 and Sm29, provides significant protection against Schistosoma mansoni in murine infection model. BMC Infect Dis. 2015;15:147. doi: 10.1186/s12879-015-0906-z . 25887456 PMC4389862

[38] El-Shabasy EA, Reda ES, Abdeen SH, Said AE, Ouhtit A. Transmission electron microscopic observations on ultrastructural alterations in *Schistosoma mansoni* adult worms recovered from C57BL/6 mice treated with radiation-attenuated vaccine and/or praziquantel in addition to passive immunization with normal and vaccinated rabbit sera against infection. Parasitol Res. 2015;114(4):1563–80. Epub 2015/03/20. doi: 10.1007/s00436-015-4341-2 . 25786393

[39] Loukas A, Tran M, Pearson MS. Schistosome membrane proteins as vaccines. Int J Parasitol. 2007;37(3–4):257–63. Epub 2007/01/16. doi: 10.1016/j.ijpara.2006.12.001 . 17222846

[40] LoVerde PT, Niles EG, Osman A, Wu W. *Schistosoma mansoni* male–female interactions. Can J Zool. 2004;82(2):357–74.

[41] Andreu Z, Yáñez-Mó M. Tetraspanins in extracellular vesicle formation and function. Front immunol. 2014;5:442. doi: 10.3389/fimmu.2014.00442 . 25278937 PMC4165315

[42] Du P, Giri BR, Liu J, Xia T, Grevelding CG, Cheng G. Proteomic and deep sequencing analysis of extracellular vesicles isolated from adult male and female *Schistosoma japonicum*. PLoS Negl Trop Dis. 2020;14(9):e0008618. Epub 2020/09/29. doi: 10.1371/journal.pntd.0008618 ; PubMed Central PMCID: PMC7521736. 32986706 PMC7521736

[43] Riner DK, Ferragine CE, Maynard SK, Davies SJ. Regulation of Innate Responses during Pre-patent Schistosome Infection Provides an Immune Environment Permissive for Parasite Development. PLoS pathogens. 2013;9(10):e1003708. doi: 10.1371/journal.ppat.1003708 24130499 PMC3795041

[44] Mukendi JPK, Nakamura R, Uematsu S, Hamano S. Interleukin (IL)-33 is dispensable for *Schistosoma mansoni* worm maturation and the maintenance of egg-induced pathology in intestines of infected mice. Parasit Vectors. 2021;14(1):70. Epub 2021/01/24. doi: 10.1186/s13071-020-04561-w ; PubMed Central PMCID: PMC7821721. 33482904 PMC7821721

[45] Mati VL, Melo AL. Current applications of oogram methodology in experimental schistosomiasis; fecundity of female *Schistosoma mansoni* and egg release in the intestine of AKR/J mice following immunomodulatory treatment with pentoxifylline. J Helminthol. 2013;87(1):115–24. Epub 2012/03/07. doi: 10.1017/S0022149X12000144 . 22390937

[46] Etewa SE, Abd El-Aal NF, Abdel-Rahman S A, El-Shafey MA. Parasitological evaluation of potential candidate vaccines in *Schistosoma mansoni*-infected mice. JPVB. 2014;6(2):23–30.

[47] Paul S, Ruiz-Manriquez LM, Serrano-Cano FI, Estrada-Meza C, Solorio-Diaz KA, Srivastava A. Human microRNAs in host-parasite interaction: a review. 3 Biotech. 2020;10(12):510. Epub 11/05. doi: 10.1007/s13205-020-02498-6 . 33178551 PMC7644590

[48] Liu J, Zhu L, Wang J, Qiu L, Chen Y, Davis RE, et al. *Schistosoma japonicum* extracellular vesicle miRNA cargo regulates host macrophage functions facilitating parasitism. PLoS pathogens. 2019;15(6):e1007817. doi: 10.1371/journal.ppat.1007817 . 31163079 PMC6548406

[49] Wang L, Li Z, Shen J, Liu Z, Liang J, Wu X, et al. Exosome-like vesicles derived by *Schistosoma japonicum* adult worms mediates M1 type immune- activity of macrophage. Parasitol Res. 2015;114(5):1865–73. Epub 2015/04/10. doi: 10.1007/s00436-015-4373-7 . 25855345

[50] Chadwick S, Kriegel C, Amiji M. Nanotechnology solutions for mucosal immunization. Adv Drug Deliv Rev. 2010;62(4–5):394–407. Epub 2009/11/26. doi: 10.1016/j.addr.2009.11.012 . 19931581

[51] Cheever AW, Jankovic D, Yap GS, Kullberg MC, Sher A, Wynn TA. Role of cytokines in the formation and downregulation of hepatic circumoval granulomas and hepatic fibrosis in *Schistosoma mansoni*-infected mice. Mem Inst Oswaldo Cruz. 1998;93:25–32. Epub 1999/01/28. doi: 10.1590/s0074-02761998000700004 . 9921320

[52] Jankovic D, Kullberg MC, Noben-Trauth N, Caspar P, Ward JM, Cheever AW, et al. Schistosome-infected IL-4 receptor knockout (KO) mice, in contrast to IL-4 KO mice, fail to develop granulomatous pathology while maintaining the same lymphokine expression profile. J Immunol. 1999;163(1):337–42. Epub 1999/06/29. . 10384133

[53] Kaplan MH, Whitfield JR, Boros DL, Grusby MJ. Th2 cells are required for the *Schistosoma mansoni* egg-induced granulomatous response. J Immunol. 1998;160(4):1850–6. Epub 1998/02/20. . 9469446

[54] Nascimento M, Huang SC, Smith A, Everts B, Lam W, Bassity E, et al. Ly6Chi monocyte recruitment is responsible for Th2 associated host-protective macrophage accumulation in liver inflammation due to schistosomiasis. PLoS pathogens. 2014;10(8):e1004282. Epub 2014/08/22. doi: 10.1371/journal.ppat.1004282 ; PubMed Central PMCID: PMC4140849. 25144366 PMC4140849

[55] Wu C, Chen Q, Fang Y, Wu J, Han Y, Wang Y, et al. *Schistosoma japonicum* egg specific protein SjE16.7 recruits neutrophils and induces inflammatory hepatic granuloma initiation. PLoS Negl Trop Dis. 2014;8(2):e2703. Epub 2014/02/20. doi: 10.1371/journal.pntd.0002703 ; PubMed Central PMCID: PMC3923719. 24551263 PMC3923719

[56] Fonseca CT, Brito CF, Alves JB, Oliveira SC. IL-12 enhances protective immunity in mice engendered by immunization with recombinant 14 kDa *Schistosoma mansoni* fatty acid-binding protein through an IFN-gamma and TNF-alpha dependent pathway. Vaccine. 2004;22(3–4):503–10. Epub 2003/12/13. doi: 10.1016/j.vaccine.2003.07.010 . 14670333

[57] Torben W, Ahmad G, Zhang W, Nash S, Le L, Karmakar S, et al. Role of antibody dependent cell mediated cytotoxicity (ADCC) in Sm-p80-mediated protection against *Schistosoma mansoni*. Vaccine. 2012;30(48):6753–8. Epub 09/20. doi: 10.1016/j.vaccine.2012.09.026 . 23000221 PMC3488153

[58] Kifle DW, Sotillo J, Pearson MS, Loukas A. Extracellular vesicles as a target for the development of anti-helminth vaccines. Emerging Top Life Sci. 2017;1(6):659–65. doi: 10.1042/ETLS20170095 33525849

[59] Xu C, Verwaerde C, Grzych J, Fontaine J, Capron A. A monoclonal antibody blocking the *Schistosoma mansoni* 28-kDa glutathione S-transferase activity reduces female worm fecundity and egg viability. Eur J Immunol. 1991;21:1801–7. doi: 10.1002/eji.1830210804 1868871

[60] Kool M, Soullie T, van Nimwegen M, Willart MA, Muskens F, Jung S, et al. Alum adjuvant boosts adaptive immunity by inducing uric acid and activating inflammatory dendritic cells. J Exp Med. 2008;205(4):869–82. Epub 2008/03/26. doi: 10.1084/jem.20071087 ; PubMed Central PMCID: PMC2292225. 18362170 PMC2292225

[61] Pradere JP, Kluwe J, De Minicis S, Jiao JJ, Gwak GY, Dapito DH, et al. Hepatic macrophages but not dendritic cells contribute to liver fibrosis by promoting the survival of activated hepatic stellate cells in mice. Hepatology. 2013;58(4):1461–73. Epub 2013/04/05. doi: 10.1002/hep.26429 ; PubMed Central PMCID: PMC3848418. 23553591 PMC3848418

[62] Wilson RA, Coulson PS. Strategies for a schistosome vaccine: can we manipulate the immune response effectively? Microb Infect. 1999;1(7):535–43. doi: 10.1016/s1286-4579(99)80093-8 10603570

