# Supplementary material for: Schistosoma mansoni egg-derived extracellular vesicles: A promising vaccine candidate against murine schistosomiasis
Source: PLoS Negl Trop Dis. 2021 Oct 13;15(10):e0009866. doi: 10.1371/journal.pntd.0009866 (PMC8544836; doi:10.1371/journal.pntd.0009866)
Supplement: S1 Text — (DOCX) [file pntd.0009866.s001.docx]

| Parasitological parameter (mean ± S.D)  Animal groups | Adult worm load | Couple worm load | Liver egg count | Intestinal egg count | Granuloma size (µm) | Granuloma number |
| --- | --- | --- | --- | --- | --- | --- |
| Infected control (I) | 30.7 ± 3.56 | 13.60 ± 2.11 | 5133.3±1808.8 | 3145.0±871.8 | 340.40 ± 97.22 | 6.3 ± 2.03 |
| Infected adjuvanted control (II)  (%Ch) | 32.3 ± 11.54  (↑5.21 ^N. S^) | 10.80 ± 4.56  (↓20.59 ^N. S^) | 7682.9±3986.6  (↑49.7 ^N. S^) | 4170.2±2360.6  (↑32.6 ^N. S^) | 311.45±30.36  (↓8.5 ^N. S^) | 4.40 ± 1.35  (↓30.16 ^N. S^) |
| Infected vaccinated (III)  (%Ch) | 16.40 ± 5.02  (↓46.58*) | 7.70 ± 3.76  (↓43.38*) | 351.90±221.9  (↓93.14*) | 214.90±118.03  (↓93.17*) | 117.0±28.23  (↓65.63*) | 1.50 ± 1.1  (↓76.19*) |
| Infected vaccinated adjuvanted (IV)  (%Ch) | 9.60 ± 3.81  (↓68.7*) | 4.20 ± 1.58  (↓69.12*) | 1574.80±584.3  (↓69.32*) | 863.0±374.01  (↓72.56*) | 141.65±20.39  (↓58.39*) | 2.90 ± 1.68  (↓53.97*) |
| Significance | p_1_<0.001*, p_2_<0.001*, P_3_=0.085 | p_1=_0.053,  p_2_<0.001^*^,  p_3_=0.009^*^ | p_1_<0.001*, p_2_<0.001*, P_3_=0.006* | p_1_<0.001*, p_2_<0.001*, P_3_=0.004* | p1<0.001*,  P2<0.001*, P3=0.474 | p_1_<0.001*, p_2_=0.018*, P_3_=0.033*. |

p value for comparing group II, III and IV to infected control group I. ^N.S^: Non significance

percentage change as compared to the infected control (%Ch).

**p_1_**: p value for comparing between **infected vaccinated** and **infected adjuvanted control, p_2_**: p value for comparing between **infected vaccinated adjuvanted** and **infected adjuvanted control, p_3_**: p value for comparing between **infected vaccinated** and **infected vaccinated adjuvanted.**

*: Statistically significant at p ≤ 0.05
